# Binary Semantic Classification Using Cortical Activation with Pavlovian-Conditioned Vestibular Responses in Healthy and Locked-In Individuals

**DOI:** 10.1093/texcom/tgab046

**Published:** 2021-07-23

**Authors:** Natsue Yoshimura, Kaito Umetsu, Alessandro Tonin, Yasuhisa Maruyama, Kyosuke Harada, Aygul Rana, Gowrishankar Ganesh, Ujwal Chaudhary, Yasuharu Koike, Niels Birbaumer

**Affiliations:** Institute of Innovative Research, Tokyo Institute of Technology, Yokohama 226-8503, Japan; ATR Brain Information Communication Research Laboratory Group, Kyoto 619-0288, Japan; Integrative Brain Imaging Center, National Center of Neurology and Psychiatry, Tokyo 187-8551, Japan; PRESTO, JST, Saitama 332-0012, Japan; Institute of Innovative Research, Tokyo Institute of Technology, Yokohama 226-8503, Japan; Wyss-Center for Bio and NeuroEngineering, Geneva CH-1202, Switzerland; Institute of Medical Psychology and Behavioral Neurobiology, University of Tübingen, 72076 Tübingen, Germany; Institute of Innovative Research, Tokyo Institute of Technology, Yokohama 226-8503, Japan; Institute of Innovative Research, Tokyo Institute of Technology, Yokohama 226-8503, Japan; Institute of Medical Psychology and Behavioral Neurobiology, University of Tübingen, 72076 Tübingen, Germany; Laboratorie d’Informatique, de Robotique et de Microelectronique de Montpellier, U. Montpellier, CNRS, 34095 Montpellier, France; CNRS-AIST Joint Robotics Laboratory, Tsukuba 305-8560 Japan; Institute of Medical Psychology and Behavioral Neurobiology, University of Tübingen, 72076 Tübingen, Germany; ALS Voice gGmbH, 72116 Mössingen, Germany; Institute of Innovative Research, Tokyo Institute of Technology, Yokohama 226-8503, Japan; Institute of Medical Psychology and Behavioral Neurobiology, University of Tübingen, 72076 Tübingen, Germany; ALS Voice gGmbH, 72116 Mössingen, Germany

**Keywords:** brain–computer interface, completely locked-in state, electroencephalography, galvanic vestibular stimulation, Pavlovian conditioning

## Abstract

To develop a more reliable brain–computer interface (BCI) for patients in the completely locked-in state (CLIS), here we propose a Pavlovian conditioning paradigm using galvanic vestibular stimulation (GVS), which can induce a strong sensation of equilibrium distortion in individuals. We hypothesized that associating two different sensations caused by two-directional GVS with the thoughts of “yes” and “no” by individuals would enable us to emphasize the differences in brain activity associated with the thoughts of yes and no and hence help us better distinguish the two from electroencephalography (EEG). We tested this hypothesis with 11 healthy and 1 CLIS participant. Our results showed that, first, conditioning of GVS with the thoughts of yes and no is possible. And second, the classification of whether an individual is thinking “yes” or “no” is significantly improved after the conditioning, even in the absence of subsequent GVS stimulations. We observed average classification accuracy of 73.0% over 11 healthy individuals and 85.3% with the CLIS patient. These results suggest the establishment of GVS-based Pavlovian conditioning and its usability as a noninvasive BCI.

## Introduction

Amyotrophic lateral sclerosis (ALS) is a neuromuscular disease that leads to loss of all motor control, including movements of eyes, face, limbs, and external sphincter in the late stage of the disease (Kiernan et al. 2011). The state, after loss of all motor control, is called the completely locked-in state (CLIS), and patients in this state lose all communication channels with their surroundings (Murguialday et al. 2011). In order to improve their quality of life by providing communication, many studies have attempted to develop brain–computer interfaces (BCIs) using electroencephalography (EEG) and functional near-infrared spectroscopy (fNIRS). A semantic “Yes/No BCI,” where the BCI directly decodes whether an individual is thinking “yes” or “no” to a particular question, has been of great interest (Kübler and Birbaumer 2008; Murguialday et al. 2011; De Massari et al. 2013; Gallegos-Ayala et al. 2014; Chaudhary et al. 2017; Okahara et al. 2018; Han et al. 2019; Khalili Ardali et al. 2019). A Yes/No BCI can enable *natural communication* between the CLIS patients and their family and caretakers, without requiring the patients to perform any other cognitive tasks unrelated to the question posed to them, such as number calculation or motor imagery, in order for the BCI to decode and understand their answers. However, the neural representations of “yes” and “no” are arguably quite different depending on questions and individual experiences and memory background. Therefore, it may be helpful to introduce a procedure that emphasizes the difference between the thought of yes/no in neural activation and in addition enhances the signal-to-noise ratio of electrocortical activity.

**Figure 1:**
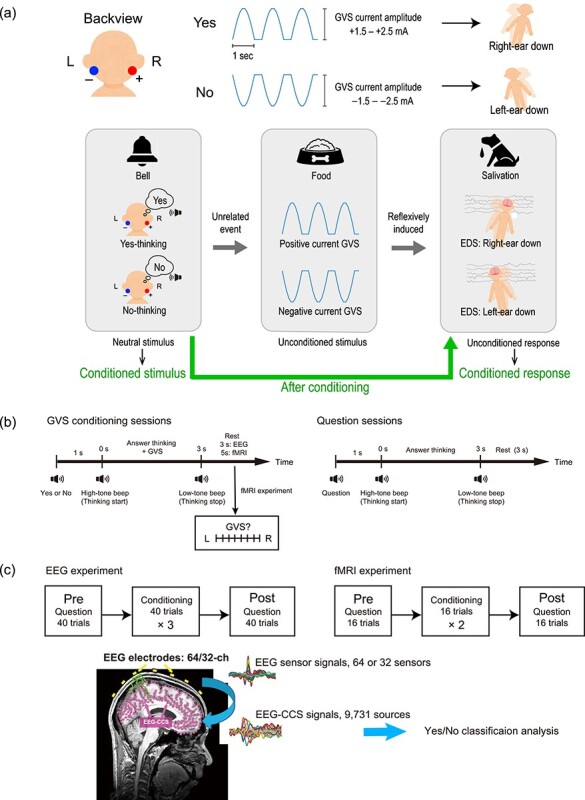
Experimental concept and paradigm. (*a*) Upper panel: Two GVS electrodes were placed behind the ears, an anode (red) electrode behind the right ear, and a cathode (blue) behind the left ear. Three positive half cycles of 0.5 Hz sine waves were provided for thought of “yes,” which resulted in right-ear-down tilt sensation. In the case of thought of “no,” 3 negative half cycles of 0.5 Hz sine waves were given to induce left-ear-down tilt sensation. Lower panel: Conceptual diagram of the thought of yes/no, GVS, and brain activity caused by EDS aligned with examples of Pavlovian conditioning. (*b*) One-trial time flow of GVS conditioning and question sessions. In both sessions, participants started thinking the answer after a high-tone beep and stopped thinking when they heard a low-tone beep. In fMRI experiments, they rated the EDS direction on a visual analog scale after the answering period of the conditioning sessions. (*c*) Session structures for EEG and fMRI experiments. Both EEG and fMRI data were used to investigate additional activated brain areas caused by the EDS after the conditioning (i.e., postconditioning sessions). We estimated EEG-CCS from EEG sensor signals, and the EEG-CCS signals were used for the yes/no classification analysis.

In order to evoke additional brain activity allowing to better distinguish the neural response to the thought of yes/no, classical conditioning, also known as Pavlovian conditioning, seems to be a promising method. As shown in the famous example (Pavlov 1927), if a dog repeatedly listens to the sound of a bell preceding feeding, the mere sound of the bell will cause the animal to salivate in anticipation of the food (Fig. 1*a*). The salivation occurs unconsciously and cannot be controlled voluntarily. Here, the important point is to associate two previously unrelated events (the conditioned stimulus (CS), in this example the sound of the bell, and the unconditioned stimulus (US), in this example the sight of food), with the unconditioned response (UR, i.e., salivation), which before conditioning is a reflexive response induced by food (the US).

For establishing Pavlovian conditioning, we introduced galvanic vestibular stimulation (GVS) as a US because equilibrium distortion sensations (EDS) such as visual rotation and tilt of the body caused by GVS are reflexive responses and expected to serve as a UR. GVS is a variation of transcranial direct current stimulation (tDCS) and excites the vestibular system that controls our body balance (Fitzpatrick and Day 2004; Utz et al. 2010). Being noninvasive, nonpainful, and safe, GVS has drawn attention not only for scientific purposes but also for applications in clinical and engineering disciplines (Maeda et al. 2005; Pan et al. 2008; Sra et al. 2017; Dlugaiczyk et al. 2019; Liu et al. 2019). Existing literature of fMRI analysis during GVS and the anatomical connections between the vestibular nuclei revealed involvement of sensorimotor-related areas (Mountcastle 1957; Lobel et al. 1998) and parietal areas to the vestibular functions (Stephan et al. 2005; Lopez et al. 2012; Reichenbach et al. 2016).

In this study, we established associations between thoughts of (i.e., covert) yes/no answers to questions and two EDSs with a Pavlovian conditioning paradigm. Using a differential conditioning paradigm as shown in Figure 1*a* (Razran 1971), two types of EDSs with different directions were constructed by altering the polarity of the current from two electrodes (one anode and one cathode) attached to the mastoids behind the ears (Utz et al. 2010), and the two EDSs were associated with thoughts of yes and no, respectively. In this paradigm, the thought of yes/no is expected to function as the neutral stimulus (NS, i.e., sound of the bell) that will become a conditioned stimulus (CS) after establishing the conditioning successfully. If the conditioning succeeds, brain activity evoked by the EDSs will become the conditioned response (CR) as does salivation. Although attempts to associate yes/no with auditory and tactile stimulation in conditioning paradigms have been made in other studies (Furdea et al. 2012; De Massari et al. 2013; Ruf et al. 2013), GVS has not been used in this paradigm. Given the reflexive nature of the EDS compared with auditory and tactile perception, EDS is expected to be easier to associate it with thought of yes/no compared with other stimulations usually with additional auditory (such as two sounds with different frequencies) or visual cues or different types of imagery. In the context of this BCI, it is the stimulus question including its semantic content asked to the patient requiring a yes or no answer together with the GVS, which constitutes the conditioned stimulus. As in the original Pavlovian experimental situation, we assume—following Pavlov—that the pairing of the neutral CS with a biologically significant stimulus (sight of food) will make an associative contingency more stable and resistant to extinction than the semantic content and the sounds of the question alone. In our case, the biologically significant stimulus consists of the two types of GVS that cause EDS, which is impossible to escape and of obvious biological significance in order to keep the body balance. This is particularly important in the case of a CLIS patient when questions may lose their power to elicit a response through an extinction process because yes or no answers are not possible anymore due to the complete paralysis and have no biologically relevant effects (i.e., no answer responses from the social environment) and thus will lose the contingency through extinction. On the subjective level, this may be experienced as disattention and loss of interest to answer any question with a yes or no response.

To anticipate our results, we found that EDS could be clearly associated with the thoughts of “yes” and “no,” which we could verify using functional magnetic resonance imaging (fMRI) where we observed clear activation in sensorimotor-related and parietal areas induced by the thought of yes/no (after association). Following the conditioning, we performed a classification analysis for the thought of “yes” versus “no” using EEG cortical current source (EEG-CCS) signals. The methodology showed appreciable performance not only with healthy participants but also with a CLIS patient.

## Materials and Methods

### Participants

Eleven healthy human participants (H1 to H11) from 23 to 55 years old (*M* = 34.5, *SD* = 12.7, 10 males and 1 female) and an ALS patient in the CLIS (P1) (male, 39 years old) participated in this study. Six participants (H1 to H6) participated in the fMRI experiment to examine brain activation difference between the pre- and postconditioning sessions. These participants then participated in an EEG experiment to examine the decoding accuracy after the conditioning. Next, we invited five naïve participants (H7 to H11) to the EEG experiment to examine the conditioning effect by comparing the yes versus no classification accuracies between the pre- and postconditioning sessions.

This is an exploratory study that aimed at formulating a basis for a clear hypothesis on the GVS conditioning effect for future studies with CLIS patients. In terms of applying this method to BCI, we intended to evaluate its effectiveness with a large effect size. In the past BCI studies, we found that even those with large effect sizes of decoding accuracies were in the range of 0.8 to 10 (Ruf et al. 2013; Fukuma et al. 2018; Irimia et al. 2018). Therefore, we calculated the effect size using our data from the six participants (H1-H6) and obtained a value of *d* = 2.26 (Mean accuracy±S.D. = 61.59 ± 5.31; Mean chance±S.D. = 51.43 ± 3.51). Then, a power analysis of the *t*-test was conducted using G*power 3.1 (Faul et al. 2007) with power set at 0.8, effect size at 2, and alpha at 0.05, resulting in a sufficient sample size of five participants. Therefore, we recorded EEG from GVS-naïve five participants (H7-H11) to examine the significance of the conditioning effect between the pre- and postconditioning sessions. The effect size calculated from the results was *d* = 1.64. Since this value is higher than Cohen’s recommendation for a large effect size (i.e., 0.8) (Cohen 1988), we assumed that a significant effect would allow a clear hypothesis for the future investigation with CLIS patients. An effect size of *d* = 2.46 was obtained using the comparison to chance level from all 11 participants.

The patient was diagnosed with bulbar ALS in 2009. He lost speech and capability to move by 2010. He has been artificially ventilated since April 2010 and is in home care. No communication with eye movements, other muscles, or assistive communication devices was possible since 2012. The study protocol for the healthy participant was approved by the ethics committee of the Tokyo Institute of Technology, Japan (Approval No. 2019017), and the protocol for the patient was approved by the Institutional Review Board of the Medical Faculty of the University of Tübingen, Germany; and the experiments were carried out in accordance with the Declaration of Helsinki. Written informed consent was obtained from each of the healthy participants before the experiment and, in the case of the patient, from the patient’s legal representative.

### GVS Procedure

The positive and negative half cycle of 0.5 Hz sine waves were created by MATLAB R2014a (The MathWorks, Inc.) and sent to the DC Stimulator Plus (neuroConn, neuroCare Group GmbH) via a digital-analog converter (NI USB-6225, National Instruments Corporation) to provide the electrical current to the two electrodes placed behind the ears. As shown in Figure 1*a*, 3 repetitions of the positive or negative half cycle wave were provided in one trial (i.e., during thoughts of “yes” or “no”), which resulted in 3-s stimulation per trial. The positive waves were provided during thoughts of yes, and the negative waves were used for the thought of no. The anode (positive) electrode was placed behind the right ear, and the cathode (negative) electrode was placed behind the left ear. Therefore, the direction of the EDS was different depending on the content of the thought; the EDS occurs toward the anode, which means persons felt a right-ear-down EDS during the thought of “yes” and a left-ear-down EDS during the thought of “no.” The absolute maximum value of the sine waves was in a range of 1.0–3.0 mA as determined by each participant’s scaling before the experiment so that she or he could recognize the direction difference of the EDS. For the CLIS patient, his sister (legal representative) decided the amplitude as 2.0 mA based on her own experience of the GVS.

### Experimental Paradigms of Classical Conditioning and Question Sessions

The experiment was conducted in the following order: a preconditioning question session, conditioning sessions, and a postconditioning question session (Fig. 1*c*). GVS was provided to the participants only in the conditioning sessions (Fig. 1*b*). Both the fMRI and EEG experiments were conducted with the participants lying in supine position with their eyes closed so as to replicate the CLIS patient’s posture. All the participants were instructed about the task sequence described below before the experiment.

In the conditioning sessions, GVS was applied to the participants when they thought “yes” and “no.” Specifically, in one trial, they first heard the spoken word “yes” or “no” and started thinking that word after they heard a high-tone beep sound. Positive GVS, for “yes” (right-ear-down distortion), and negative GVS, for “no” (left-ear-down distortion), were given to the participants 1 s after the high-tone beep. The participants were instructed to stop thinking after 3 s when hearing a low-tone beep. In the case of the fMRI experiments, we asked the participants to report their perceived direction of EDS in each trial (Fig. 1*b*, left panel). The number of trial repetitions in one session varied between the EEG and fMRI experiments (See sections *fMRI Experiment* and *EEG Experiment*). We confirmed the effect of the conditioning by checking after each conditioning session whether the participants could easily or spontaneously associate the two types of EDS with the thoughts of “yes” and “no” answers. Precisely, after the training session, we confirmed that the participants could remember the difference of EDS between the thoughts of yes and no.

In the question sessions, the participants thought “yes” or “no” as an answer for an auditorily presented question in the absence of GVS. The question was randomly selected from 23 pairs of yes and no questions shown in Supplementary Table 1. All the questions were simple, and the answers were known to the participants and experimenters. The questions used for the CLIS patient in the EEG experiment were personal and chosen by his family, and the patient knew the answers to the questions according to family’s information. The experiment with the patient was performed at the patient’s home. The same beep sounds as in the conditioning sessions were used to provide starting and stopping cues.

The high- and low-tone beep sounds were created by extracting a portion of the “burn_failed.wav,” a standard tone in the windows OS at sampling rates of 25 000 Hz and 8000 Hz, respectively. The “Yes” and “No” words and questions for the patient were recorded by the patient’s legal representative, whereas the words for the healthy participants were synthesized using the Text-to-Speech function in the Macintosh OS.

### fMRI Experiment

Six of the eleven healthy participants (H1–H6) participated in the fMRI experiment. The fMRI experiment consisted of two conditioning sessions and two question sessions including one preconditioning session and one postconditioning session (Fig. 1*c*). The auditory stimuli were presented to the participants via MRI-compatible earphones (KMR-512(S), KOBATEL Corporation) in an MRI scanner. In a conditioning session, 8 yes and 8 no auditory stimuli (i.e., 16 trials in total) were provided in random order. After the thought period, a visual analog scale asking the direction of the EDS was displayed (see Fig. 1*b*), and the participants answered it using an MRI-compatible trackball mouse (HHSC-TRK-2, Current Designs Inc.). In a question session, 8 yes and 8 no questions were randomly selected from the list in Supplementary Table 1 and presented. The participants thought “yes” or “no” after the high-tone beep. The experimental software used for the conditioning and question sessions, such as presenting the auditory stimuli and sending the sine waves for GVS, were all written in MATLAB R2018b, using the Psychophysics Toolbox extensions (Brainard 1997; Pelli 1997; Kleiner et al. 2007).

A 3 T Magnetom Prisma MRI scanner equipped with a 32-channel array coil (Siemens) was used for the functional and anatomical MRI acquisition. During the experiment, the participants lay on the scanner bed in a supine position with eyes closed to replicate the posture of the CLIS patient. In the conditioning sessions, they opened their eyes when they heard the low-tone beep sound to indicate the direction of the GVS. The visual analog scale was displayed on a 32-inch BOLDscreen (Cambridge Research Systems) and presented to the participants through a mirror that was mounted over their faces. Functional data were acquired with a *T*2^*^-weighted gradient-echo, echo-planar imaging sequence using the following parameters: repetition time (TR) = 2.5 s; echo time (TE) = 30 ms; flip angle (FA) = 80°; field of view (FOV) = 212 × 212 mm; matrix size = 64 × 64; 40 slices; slice thickness = 3.2 mm. In the conditioning sessions, we did not fix the time for the participants to report the direction of GVS using the trackball mouse after the thoughts of yes and no. In the question sessions, the time period required for presenting questions was different from one question to another. A brain fMRI volume refers to one complete 3D image of the brain. The time taken to record one volume is TR (i.e., repetition time). Due to difference in the response time by our participants (which was not fixed) and due to differences in the length of questions presented to the participants, the length of the fMRI sessions and hence the number of brain volumes varied across sessions and participants. A 3D anatomical image was acquired using an MPRAGE *T*1-weighted sequence (TR = 1900 ms; TE = 2.52 ms; FA = 9°; FOV = 256 × 256 mm; matrix size = 256 × 256; 192 slices; slice thickness = 1.2 mm).

### fMRI Data Analysis

fMRI data analysis was performed using SPM12 (Wellcome Department of Cognitive Neurology; http://www.fil.ion.ucl.ac.uk/spm) running on MATLAB R2016b for individual participant analysis. Statistical analyses were performed using a general linear model (GLM) after the standard preprocessing (i.e., spatial realignment to the mean EPI image, slice timing corrections, coregistration of a bias-corrected *T*1-weighted anatomical image to the realigned images, normalization to the Montreal Neurological Institute (MNI) standard brain, and smoothing with a full-width spatial Gaussian kernel of 8 mm at half maximum). The yes/no thought periods were modeled using boxcar functions and convolved with the hemodynamic response function. After the model parameters estimation, statistical parametric maps for each participant were created using four conditions: Yes > No (in preconditioning), No > Yes (in preconditioning), Yes > No (in postconditioning), and No > Yes (in postconditioning) with *P* < 0.001 (uncorrected for multiple comparisons). One-sample *t*-tests were conducted for the group analysis using the four contrasts from the six participants by setting the regions of interest (ROIs). Based on the existing literature on GVS and galvanic vestibular system (Mountcastle 1957; Lobel et al. 1998; Stephan et al. 2005; Lopez et al. 2012; Reichenbach et al. 2016), we fixed the ROI to sensorimotor-related areas [postcentral gyrus, precentral gyrus, and supplemental motor areas (SMA)] and parietal areas (angular gyrus, precuneus, parietal operculum, supramarginal gyrus, and superior parietal lobule) using maximum probability tissue labels derived from the Neuromorphometric atlas (provided by Neuromorphometrics, Inc. http://Neuromorphometrics.com) as implemented in SPM12.

### E‌EG Experiment

The EEG experiment consisted of three consecutive conditioning sessions with 40 trials, followed by one question session (postconditioning session) with 40 questions (Fig. 1*c*). For the participants H7–H11, preconditioning session with 40 questions was performed before the conditioning sessions. The auditory stimuli were presented using stereo speakers. In one conditioning session, 20 yes and 20 no auditory stimuli were presented in random order. In a question session, 20 yes and 20 no questions were randomly selected from the list in Supplementary Table 1 and presented. As in the fMRI experiment, the participants thought “yes” or “no” after the high-tone beep and were instructed to stop the thought when they heard the low-tone beep. The experimental program was written using MATLAB R2014b.

For the healthy participants, EEG signals were recorded from 64-channel active electrodes placed according to the extended international 10–20 system layout using the ActiveTwo system and the ActiView software (BIOSEMI) with a sampling rate of 512 Hz. The 64-channel locations are Fp1, AF7, AF3, F1, F3, F5, F7, FT7, FC5, FC3, FC1, C1, C3, C5, T7, TP7, CP5, CP3, CP1, P1, P3, P5, P7, P9, PO7, PO3, O1, Oz, Iz, POz, Pz, CPz, Fpz, Fp2, AF8, AF4, Afz, Fz, F2, F4, F6, F8, FT8, FC6, FC4, FC2, FCz, Cz, C2, C4, C6, T8, TP8, CP6, CP4, CP2, P2, P4, P6, P8, P10, PO8, PO4, and O2. During the experiment, they lay on a bed in a supine position in an electrically shielded soundproof room (AMC-3515, O’HARA & Co., Ltd) with eyes closed so as to replicate the CLIS patient’s posture.

For the patient, EEG signals were recorded from 32-channel active electrodes using a BrainAmp DC amplifier and actiCAP snap (Brain Products GmbH) with a sampling rate of 500 Hz. The 32-channel locations are Fp1, Fz, F3, F7, FT9, FC5, FC1, C3, T7, TP9, CP5, CP1, Pz, P3, P7, O1, Oz, O2, P4, P8, TP10, CP6, CP2, Cz, C4, T8, FT10, FC6, FC2, F4, F8, and Fp2. The patient also lay on a bed at his home in a supine position, which he usually stays in. His eyes were closed (only manual opening is possible in CLIS). We did not perform preconditioning session by considering the burden of the patient.

### E‌EG Data Preprocessing

EEG raw data were loaded into MATLAB using the EEGLAB toolbox (https://sccn.ucsd.edu/wiki/EEGLAB) (Delorme and Makeig 2004). The loaded data were band-pass filtered between 0.5 Hz and 40 Hz, and epoched in reference to the onset of GVS that started 1 s after the high-tone beep sound that indicated the start of the imagery. Each epoch had a duration of 6 s, 2 s of preonset and 4 s of postonset. The epoched 3-dimensional matrix (i.e., channel × timepoints × trials) were saved with other information that was required for the following EEG-CCS estimation.

### E‌EG-CCS Estimation

We examined whether the thoughts of yes and no could be discriminated using the EEG-CCS signals. EEG-CCS was estimated using the distributed source localization methods called Variational Bayesian Multimodal EncephaloGraphy method (VBMEG) toolbox (ATR Neural Information Analysis Laboratories; http://vbmeg.atr.jp/?lang=en) (Sato et al. 2004). The coordinate positions of 9731 vertices are defined on the cortical surface of the MNI standard brain (Fig. 1*c*, pink dots in the left-bottom panel), and time series of the vertices (i.e., EEG-CCS) were estimated from the 64-channel EEG sensor signals (32-channels for the CLIS patient) using a hierarchical Bayesian framework (Sato et al. 2004). A *T*1-weighted MRI anatomical image is often used to create an individual brain model for each person. In this study, however, considering the difficulty of obtaining MRI images from patients in the CLIS, we used an MNI standard brain model and a lead-field matrix that is provided by the toolbox also for the healthy participants, instead of using their individual MRI images. The brain model includes XYZ coordinates of 9731 vertices, and the lead-field matrix is a forward filter to calculate EEG signals from the defined EEG-CCS signals based on sulci and gyri geometry and difference of electrical conductivities between scalp, skull, and cerebrospinal fluid (CSF). A Bayesian framework was used to estimate an inverse filter that calculates EEG-CCS signals from EEG sensor signals. We used default parameters defined by VBMEG throughout the EEG-CCS estimation. The inverse filter was estimated using all the trial data including both answers with the Bayesian activation prior as “uniform,” and EEG-CCSs were calculated by applying the preprocessed EEG data to the inverse filter. The EEG-CCSs were estimated for the whole cortex.

### Yes/No Classification Using EEG-CCS

We performed a binary classification analysis between the thoughts of yes and no using the estimated EEG-CCS and Sparse Logistic Regression (SLR) toolbox version 1.2.1 alpha (Yamashita et al. 2008) (ATR Computational Neuroscience Laboratories; https://bicr.atr.jp/∼oyamashi/SLR_WEB.html). Since locations of current source vertices are assigned to the cortical areas according to the automated anatomical labeling atlas (AAL) (Tzourio-Mazoyer et al. 2002) in the toolbox, we can select EEG-CCS signals to be used for the classification analysis based on anatomically defined areas. In order to examine the conditioning effect on the classification accuracy, it is desirable to use signals from all areas of six sensorimotor-related areas (i.e., left and right precentral, postcentral, and SMA) and twelve parietal areas (left and right superior parietal gyrus, inferior parietal gyrus, supramarginal gyrus, angular gyrus, precuneus, and paracentral lobule). However, the total number of vertices in the areas are 2648 that will not provide high accuracy due to overfitting. On the other hand, there are countless combinations of areas to select some of the 18 areas, and the aim of this study is not developing an algorithm but proposing the concept of the GVS conditioning to enhance binary semantic classification performance. Therefore, at first, we examined the conditioning effect using the average time series of each of the 18 anatomical areas. Next, to see the possibility to achieve higher accuracies, we performed a classification analysis using unaveraged signals, by selecting anatomical areas on a trial-and-error basis, with sensorimotor-related areas as the priority. For participants except H2, H3, H5, and H7, in cases where the classification accuracy was less than 60% when using areas from the six areas only, other areas were additionally selected on a trial-and-error basis by referring to activation areas observed by individual fMRI analysis results of participants H1–H6. The mean classification accuracy was calculated using 20-times 20-fold cross-validations for each pre- and postconditioning session (i.e., using 40 trials data consisting of 20-yes and 20-no). Statistical analyses were performed using a two-sample *t*-test. Chance levels were calculated in a data-driven manner by randomizing the dataset labels of the postconditioning session in order to test for significance more rigorously.

## Results

### Association between the EDSs and Thought of Yes/No

Reports from all participants who performed the fMRI experiment (H1–H6) revealed that they recognized the GVS directions in all trials without any inconsistency in the conditioning sessions. All of the participants (H1–H11) reported that they felt their own EDSs even in the absence of GVS in the question sessions. The type of EDS varied from participant to participant, with some reporting that their body was being pushed from one side or pulled, their vision was rotating, or they felt as if the center of their body was rotating.

### Activations in the Sensorimotor-Related and Parietal Areas during Thoughts of Yes and No after the GVS Conditioning

**Figure 2:**
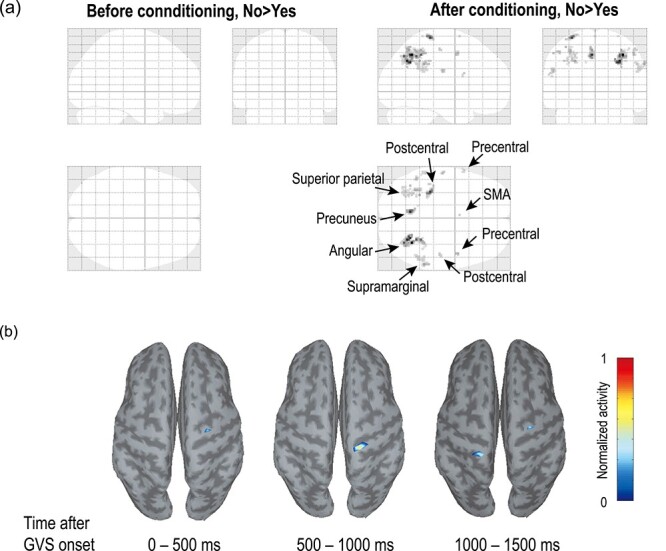
Differential brain-activity areas during the pre- and postconditioning sessions. (*a*) Results of an fMRI group analysis displaying areas of higher activity during “no” with respect to “yes” before and after conditioning. Each map represents the difference between the thoughts of yes and no, and the ROIs were set to sensorimotor-related (the postcentral, the precentral gyri, and the SMA on the left and right hemispheres) and parietal (the angular gyrus, precuneus, parietal operculum, supramarginal gyrus, and superior parietal lobule on the left and right hemispheres) areas. The activation areas projected on standard transparent brains are presented in coronal, axial, and sagittal views. The areas are statistically significant without multiple comparison corrections, uncorrected *P* < 0.001. (*b*) Brain topographical maps showing averaged EEG-CCS activation during the thought of “no” in the postconditioning session in participant H3. The dark gray and the light gray areas represent sulci and gyri, respectively. The high activation area was located in the postcentral gyrus around 500–1000 ms after the “expected” GVS onset when GVS was supposed to be applied.

Figure 2*a* shows the results of the fMRI group analysis depicting the difference in brain activity during the thought of yes and no in participants H1–H6. Although the laterality differences in activity varied depending on the participants, the group analysis revealed significant difference mainly in the angular gyrus, precuneus, and postcentral gyrus with a higher activation during “no” with respect to “yes” (*T* = 19.63, 15.73, and 15.31 for the areas, respectively, degrees of freedom = 5 and *P* < 0.001 for all, uncorrected, Table 1). The difference was observed not in the preconditioning session but in the postconditioning session only, and no significant higher activation difference was observed during “yes” with respect to “no” (*T* = 1.48, degrees of freedom = 5, *P* = 0.095, uncorrected, for the highest activation in the precentral gyrus right). Table 1 shows detailed information of the significant activity differences in the selected areas.

**Table 1:** Brain areas showing increased differential activation between thoughts of yes and no during the postconditioning session from the fMRI analysis

Name of area	MNI coordinates	*T*-values
Angular gyrus right	[34, −58, 46]	19.63
Precuneus left	[−6, −60, 46]	15.73
Postcentral gyrus left	[−32, −34, 68]	15.31
Supramarginal gyrus right	[62, −42, 34]	9.53
Superior parietal lobule left	[−34, −52, 50]	9.14
Precentral gyrus right	[48, 2, 48]	8.70
Postcentral gyrus left	[−56, −18, 24]	8.63
Angular gyrus left	[−50, −52, 50]	8.17
Superior parietal lobule left	[−30, −56, 38]	7.82
Supramarginal gyrus left	[−56, −40, 48]	7.69
Postcentral gyrus right	[50, −22, 54]	7.43
Precentral gyrus left	[−60, 10, 22]	7.34
Angular gyrus left	[−28, −68, 36]	7.15
Supramarginal gyrus left	[−42, −36, 38]	6.99
Angular gyrus left	[−38, −68, 40]	6.85
Supramarginal gyrus left	[−58, −44, 28]	6.79
SMA left	[−2, 4, 70]	6.74
Precentral gyrus left	[−58, 8, 18]	6.57
Precentral gyrus left	[−62, 8, 20]	6.56

The statistical analysis was performed by restricting the ROI to the left and right postcentral and precentral gyri, and the SMA, angular gyrus, precuneus, parietal operculum, supramarginal gyrus, and superior parietal lobule, respectively, with no multiple comparison correction (uncorrected *P* < 0.001). XYZ coordinates of the centroid of the activated areas were defined according to Montreal Neurological Institute (MNI) coordinate system.

Next, we examined the brain areas of strong activity in the EEG-CCS during the postconditioning session as well. High activation tended to be observed in the postcentral gyrus as shown in Figure 2*b*, although the exact location and the intensity of the activity varied among participants.

### GVS Conditioning Improves on the Yes/No Classification Using EEG-CCS Signals

The classification accuracy using the average signals of the 18 anatomical areas in sensorimotor-related and parietal areas was significantly higher in the postconditioning session (mean ± S.D. = 63.87 ± 7.96) than in the preconditioning session (mean ± S.D. = 53.09 ± 4.86) (*T* = 3.19, *P* = 0.03, effect size *d* = 1.64, five participants). The results from the 11 participants also showed that the mean accuracy significantly exceeded the mean chance level (postconditioning session: mean ± S.D. = 62.63 ± 6.39; chance level: mean ± S.D. = 50.37 ± 3.00; *T* = 5.91, *P* = 1.49e−04, effect size *d* = 2.46).

**Figure 3:**
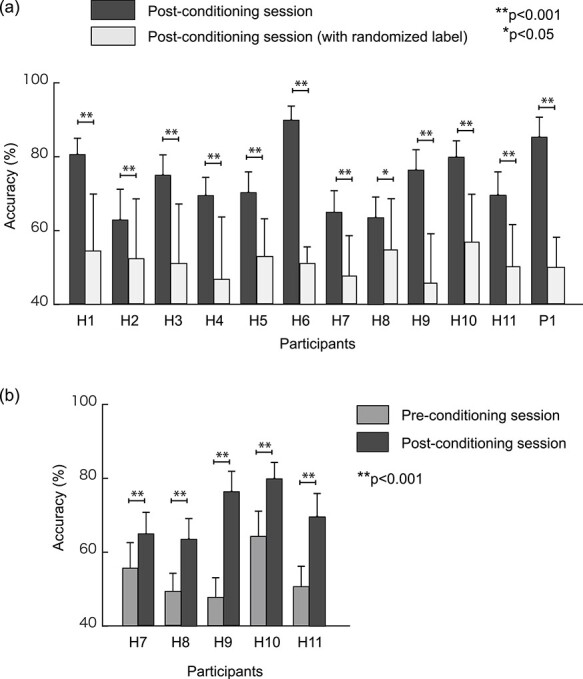
Yes/No classification accuracies using EEG-CCS for healthy participants and a CLIS patient. (*a*) Comparisons between the postconditioning session and chance level. Dark gray and light gray bars represent classification accuracies using question session data after the conditioning (i.e., postconditioning session), and postconditioning session with randomized label (i.e., chance level), respectively. (*b*) Comparisons between the pre- and postconditioning sessions. Dark-gray and gray bars represent classification accuracies using question session data in the postconditioning session, and a question session data in the preconditioning session, respectively, for the last 5 participants. The first six participants did not perform a preconditioning session. For both figures, the mean accuracies were calculated from 20-time 20-fold cross-validation analysis, and statistical significance was evaluated using two-sample *t*-test. Error bars represent standard deviations.

Next, we investigated the possibility of obtaining higher accuracies by using unaveraged signals in anatomical areas selected on a trial-and-error basis. Figures 3*a*, *b* show the comparisons between individual mean accuracies of the postconditioning session and the chance level and the individual mean accuracies of the preconditioning session, respectively. As shown in Figure 3*a*, our methodology showed the mean accuracies significantly higher than chance level with all the participants (H1–H11) and the CLIS patient (P1). The mean accuracy ($\pm$standard deviation) across the participants and the patient was 74.0 $\pm$ 8.7%. In addition, as shown in Figure 3*b*, we also confirmed that the mean accuracies of the postconditioning session were significantly higher than those of the preconditioning for all participants (H7–H11). Table 2 summarizes brain areas used for the yes/no classification analysis by each participant. The postcentral gyrus contributed to the significant accuracy in all participants, four of the participants (H2, H3, H5, and H7) showed significant accuracies using areas in sensorimotor-related areas only, and the other participants required other areas such as parietal areas: the right angular gyrus, the left calcarine, the bilateral medial superior frontal gyrus (mSFG), the bilateral cuneus, the right inferior parietal gyrus (IPG), the left precuneus, the left inferior temporal gyrus, and the right superior parietal gyrus (SPG). Our methodology also showed high significant classification accuracy (85.3$\pm$5.4%) for the CLIS patient using sensorimotor-related areas as well as the healthy participants.

**Figure 4:**
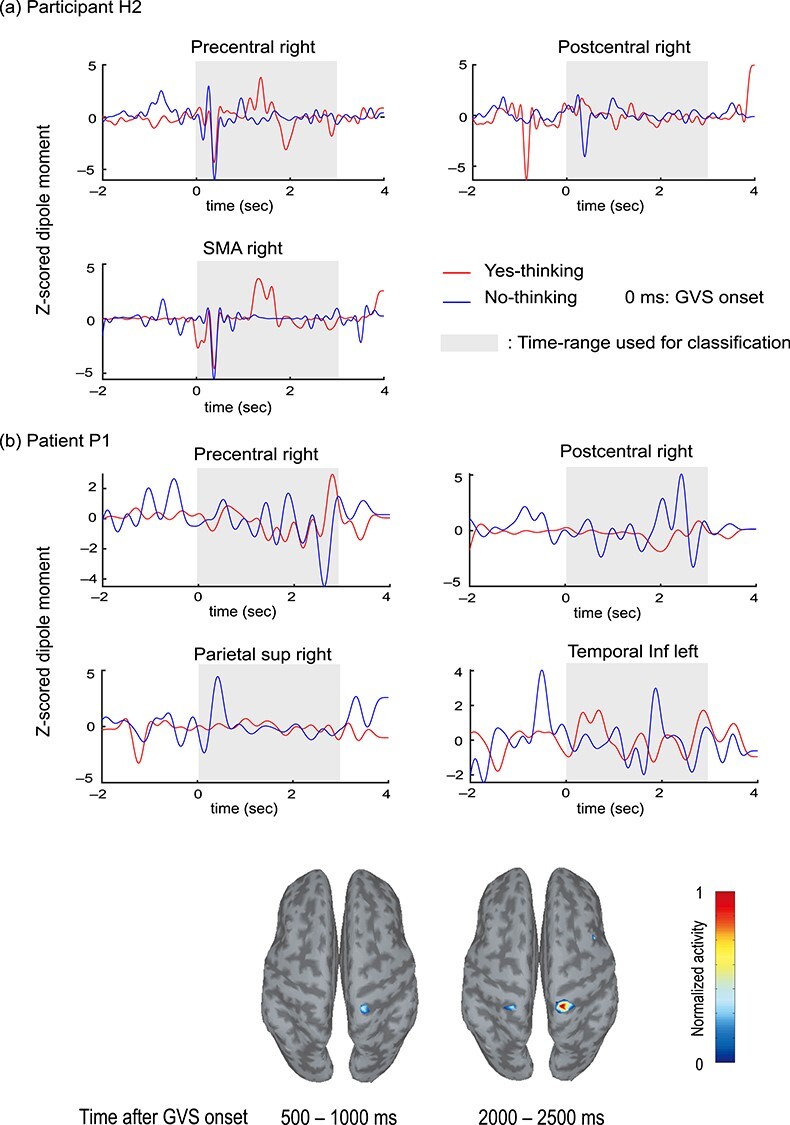
EEG-CCS activation pattern comparison between thoughts of yes and no. Mean time-series activation patterns of representative participant H2 (*a*) and patient P1 (*b*) were plotted. Red and blue lines represent the thoughts of yes and no, respectively. The gray-shaded time range was used for the classification. All time-series signals were band-pass filtered for the range of 5–15 Hz. The brain topographical maps at the bottom panel show averaged EEG-CCS activation during the question session of patient P1. The dark gray areas represent sulci and the light gray areas represent gyri. The highest activation area located in the postcentral gyrus around 2000–2500 ms after the GVS onset when GVS was supposed to be applied.

To visualize the activation difference between the thoughts of yes and no in EEG-CCS, the temporal patterns from representative participant H2 and patient P1 are shown in Figure 4. We found that the healthy participants tended to show activation difference between the thoughts of yes and no in sensorimotor-related areas mainly within 1 s after the expected GVS onset (note that GVS was not actually presented). On the other hand, the CLIS patient showed high activity in the postcentral gyrus in the EEG-CCS topographical map as well as the healthy participants, but the temporal peak differences between yes and no especially in the precentral and postcentral gyri were observed later in time than in the healthy participants (i.e., around 2 s after the GVS onset).

## Discussion

Based on the hypothesis that GVS, which evokes reflexive EDS, is suitable for Pavlovian conditioning, the present study tested whether EDS can be conditioned to thoughts of “yes” and “no.” In addition, we also investigated whether sensorimotor-related and parietal areas were activated by the EDS contingent with the thoughts of yes and no using fMRI and examined whether EEG-CCS in those areas improved the accuracy of predicting “yes” and “no” covert (i.e., cognitive) responses. All healthy participants reported that the EDS was induced by the thoughts of yes and no after conditioning, and not only fMRI but also EEG-CCS analyses confirmed that the difference in brain activity between “yes” and “no” was especially found in the postcentral gyrus. Furthermore, prediction of cognitive “yes” and “no” responses using EEG-CCS signals after conditioning achieved significant classification accuracies not only with all the healthy participants but also with the CLIS patient (73.0 $\pm$ 8.3% for healthy, 85.3 $\pm$ 5.4% for the CLIS patient).

### Activation Areas Induced by the EDS in the Postconditioning

Consistent with the classical conditioning literature (Razran 1971), we verified that after conditioning, EDS occurred as expected even in the absence of GVS and was associated with the thoughts of yes and no. GVS evokes EDS as UR to keep the body balance in equilibrium against gravity and constitutes an ideal biologically relevant US without producing negative emotional side effects of painful or unpleasant USs (Utz et al. 2011).

As shown in Figure 2, comparing the results between the pre- and postconditioning sessions using the fMRI group analysis, it was confirmed that the brain activity indicating the difference between “yes” and “no” was increased in the sensorimotor-related and parietal areas, especially in the angular gyrus, precuneus, and postcentral gyrus. This finding is not only consistent with previous fMRI (Lobel et al. 1998; Stephan et al. 2005), TMS (Reichenbach et al. 2016), and GVS (Lopez et al. 2012; Ganesh et al. 2018) research, but also with an anatomical study in cats showing that the vestibular nuclei project to the primary somatosensory cortex via the thalamus (Mountcastle 1957). Therefore, the present results, in which vestibular-related activity was observed even while the GVS was not given, may indicate successful conditioning.

In addition, it is noteworthy that the relevant area was recognized by the EEG-CCS topographical maps as well as the fMRI analysis, which may support the validity of the significant classification accuracies in this study. In the EEG-CCS classification analysis, not only the postcentral gyrus but also the precentral gyrus and SMA showed high differentiation in some participants. Since the precentral gyrus and SMA are included in the representative areas related to motor control, conditioned reflexes evoked by EDS may include neural activity related to motor control as well as sensory perception.

### Contribution of Other Areas to the Yes/No Classification

Conditioning induced differential activity in areas other than sensorimotor-related areas used in the EEG-CCS classification analysis in some participants. The areas were the right angular gyrus, the left calcarine, the bilateral mSFG, the bilateral cuneus, the right IPG, the left precuneus, the left ITG, and the right SPG. Among these areas, the angular gyrus, IPG, precuneus, and SPG are included in parietal areas, which have been reported to be activated by GVS in several studies (Stephan et al. 2005; Lopez et al. 2012; Reichenbach et al. 2016; Ganesh et al. 2018).

Regarding the involvement of the other areas (i.e., calcarine, mSFG, cuneus, and ITG), the calcarine cortex (used by H4), the bilateral cuneus (used by H6 and H11), and the ITG (used by P1) have been reported to be involved in visual processing. The calcarine cortex is located in the primary visual cortex, and an EEG study has indicated its involvement in visuospatial attention (Di Russo et al. 2003). The cuneus is also located in the primary visual cortex and receives visual information from the primary visual area V1 (Vanni et al. 2001), and the ITG is reported to be involved in visual processing via the inferior occipital gyrus (Kastner and Ungerleider 2000). The mSFG used by H6 and H9 is involved in motor control since it includes SMA and presupplementary motor area. These findings suggest that all areas used for the classification along with the sensorimotor-related areas in all participants are involved in sensorimotor integration and visual processing, contributing to the classification of the different EDS between the thoughts of yes and no.

**Table 2:** Brain areas used in the yes/no classification for the participants and the patient

ID	Areas used for the classification analysis
H1	Postcentral right, SMA left, Angular right
H2	Postcentral right, Precentral right, SMA right
H3	Postcentral right and left, Precentral right and left, SMA right and left
H4	Postcentral left, Precentral left, SMA left, Calcarine left, mSFG right
H5	Postcentral left, Precentral left, SMA right
H6	Postcentral right, SMA left, Cuneus right, mSFG left and right
H7	Postcentral left, Precentral left, SMA left
H8	Postcentral left, Precentral left, IPG right
H9	Postcentral left, SMA right, mSFG left and right
H10	Postcentral right, SMA left, IPG right, Precuneus left
H11	Postcentral right, SMA right, ITG left, Cuneus left
P1	Postcentral right, Precentral right, SPG right, ITG left

All of them showed EEG-CCS signals from the postcentral gyrus.

SMA, supplementary motor area; IPG, inferior parietal gyrus; mSFG, medial superior frontal gyrus; ITG, inferior temporal gyrus; SPG, superior parietal gyrus.

### Efficacy of the GVS for Differential Conditioning

The significant accuracies in the yes/no classification revealed the effectiveness of the GVS for differential conditioning both for the healthy and the CLIS participants.

A similar conditioning approach has been used in yes/no BCI studies with healthy participants, a CLIS patient, and two LIS (locked-in-state with intact eye movements) (Furdea et al. 2012; De Massari et al. 2013; Ruf et al. 2013). In one of these studies with CLIS and LIS patients, only thought of “yes” as a CS and a tactile sensation of electrical stimulation over the left thumb as a US were used (De Massari et al. 2013), but mean accuracies were around chance level though in some sessions yes/no classification accuracies of 70% were reached. The remaining studies tried a differential paradigm with healthy participants using pink and white noises as USs to condition thoughts of yes and no as CSs, and at most around 70% accuracies were observed. Considering that mean accuracy across the 11 participants and the CLIS patient in our study was 74.0$\pm$8.7% ($\pm$standard deviation) and the accuracies may be further improved by expanding and optimizing the areas used for classification, the current results suggest efficacy of GVS for differential conditioning to create an association between the thoughts of yes and no and the EDS. The USs used in the previous studies (i.e., tactile sensation by electrical stimulation and auditory stimuli) may not activate a stable and/or intensive and biologically relevant response compared with galvanic vestibular responses. In GVS, the participants felt not only the sensation on the skin caused by the electrical stimulation but also the EDS that adds to the unconditioned response complex. This might have been the key to the success of the current study as hypothesized.

### Differences between Healthy Participants and a CLIS Patient

Our methodology also showed high yes/no classification accuracy of 85.3% in the CLIS patient using mainly the sensorimotor-related areas as shown in the healthy participants. Under the plausible assumption that the galvanic vestibular function remains unchanged by the disease, we expected the GVS conditioning to be also effective for the patient.

The brain areas that provided the high accuracy included the postcentral gyrus right and the precentral gyrus right that are known to be crucial sensorimotor areas. Although we did not ask the patient if he felt the EDS because of the communication deficit, the brain activity patterns in these areas shown by EEG-CCS (Fig. 4) and the high accuracy suggested intact galvanic vestibular function in the patient. In addition, we observed a notable difference between healthy participants and the CLIS patient, which may be important for future BCI developments for CLIS: While healthy participants differentiated between the two requested responses for the thoughts of yes and no within 1 s after the expected GVS onset, the patient showed a delayed response (around 2 s after the onset). This might suggest delayed neural response in the CLIS patient, which may be correlated with the dominance of slow EEG frequencies in CLIS patients (Hohmann et al. 2018; Malekshahi et al. 2019; Maruyama et al. 2021). Also, the patient involved in this study shows a dominant slow EEG of 2–4 Hz during waking hours, compared with the dominant 10 Hz frequency in healthy people, which may indicate lower arousal and/or slower cognitive processing.

### Toward Clinical Application: Critical Comments

We need to address the following issues to develop a practical application based on this methodology. At first, identification of brain areas used for the classification should be optimized. In this study, we aimed to clarify the efficacy of the GVS conditioning. Therefore, we used brain areas primarily from sensorimotor-related areas and did not optimize the accuracies using other areas. Despite significant accuracies, there may be better area combinations for each participant. To develop a practical application, as a next step, we are going to develop an algorithm to select optimal brain areas for each participant using EEG-CCS.

In the process of the algorithm development, the second issue, reproducibility, also needs to be considered for online classification. The significantly high accuracies revealed the physiological stability of topographically specific brain responses across participants and between fMRI and EEG-CCS, and they also suggest the possibility of the classical conditioning paradigm as a robust and reproducible basis for BCI development (Birbaumer 2006). Since we calculated yes/no classifiers using data selected from a session from the same day as the data used for test, we need to investigate further the effectiveness of classification in those areas using data from other days. Reproducibility is the most challenging problem of BCIs based on machine learning. Recent developments of machine learning techniques have provided powerful means to extract detailed information hidden in the brain data, especially for noninvasively recorded brain activity that consists of a complex combination of physiological processes. However, when it comes to applications of online BCIs, such detailed information extracted from experimental data rarely shows reproducibility, and it is difficult to obtain high classification accuracies with a classifier calculated with data from another day.

Among the BCIs that try to extract covert thoughts from neural activity, a paradigm based on event-related desynchronization (ERD) occurring with motor imagery has shown reliable results (Pfurtscheller et al. 1996; Pfurtscheller and Neuper 2001). The “thinking” paradigm used here constitutes a comparable approach. Considering that neural activity relative to ERD can also be observed in fMRI (Halder et al. 2011), the key to develop a reliable BCI will be the use of a paradigm that shows robust differential activation in most noninvasive brain measures such as EEG, fMRI, and NIRS. Since our results suggest the physiological stability of the CRs in terms of topographical brain responses between fMRI and EEG-CCS, the challenge will be to prove the efficacy of classifiers calculated from different days.

The third issue is optimization of the conditioning learning process. The most effective procedures to secure stable associations between CS-CR need to be varied systematically. It is usually assumed that the association between CS and US will become stronger as the number of conditioning trials increases. However, a few participants reported decreasing EDS (i.e., perception) as the number of conditioning trials increased, indicating habituation. That might be due to the low electrical currents used, and on the other hand, the brain activation still might occur even if participants do not perceive the EDS consciously. In any case, we may need to optimize and individualize the timing, frequencies, and strength of GVS to keep the conditioning effect. Even though activation areas differ between participants, observing the regional transitions of activation before, during, and after conditioning could provide insights into the process of learning.

## Supplementary Material

SupplementaryMaterials_tgab046

## Notes

We deeply thank the CLIS patient, his family, and the healthy participants. *Conflict of Interest:* N.Y., G.G., and Y.K. have a pending patent regarding the method demonstrated in this study (Japanese Patent application number: 2020-108 549).
