# Supplementary material for: Binary Semantic Classification Using Cortical Activation with Pavlovian-Conditioned Vestibular Responses in Healthy and Locked-In Individuals
Source: Cereb Cortex Commun. 2021 Jul 23;2(3):tgab046. doi: 10.1093/texcom/tgab046 (PMC8382900; doi:10.1093/texcom/tgab046)
Supplement: SupplementaryMaterials_tgab046 [file supplementarymaterials_tgab046.pdf]

Supplementary table S1. Questions used in the fMRI and EEG experiments during question sessions for the healthy participants. Answers for the questions No. 20, 22, 23 were different depending on the participants.

No.	Questions for Yes	Questions for No
1	One hour has 60 minutes?	One hour has 90 minutes?
2	Does one week consist of 7 days?	Does one week consist of 5 days?
3	Is this year xxx? *1	Is this year 1990?
4	Is this Japanese year “Reiwa”?	Is this Japanese year “Taisho”?
5	Is this month xxx? *1	Is this month May?
6	Is noon 12 o’clock?	Is noon 7 o’clock?
7	Is August in summer?	Is December in summer?
8	Is January in winter?	Is June in winter?
9	Is one meter equal to 100 centimeters?	Is one meter equal to 70 centimeters?
10	Does one year consist of 12 months?	Does one year consist of 9 months?
11	Is Mt. Fuji the highest in Japan?	Is Mt. Takao the highest in Japan?
12	1 + 1 = 2?	1 + 1 = 5?
13	2 x 5 = 10?	2 x 5 = 13?
14	10 + 7 = 17?	10 + 7 = 21?
15	Do you speak xxx? *1	Do you speak xxx? *1
16	Do you live in xxx? *1	Do you live in xxx? *1
17	Does the English alphabet consist of 26 letters?	Does the English alphabet consist of 30 letters?
18	Are apples fruits?	Are apples vegetables?
19	Is the earth a sphere?	Is the earth a cube?
20	Are you a male? / Are you a female?	
21	Are you over 20 years old?	Are you under 20 years old?
22	Do you have a driving license? / Don’t you have a driving license?	
23	Have you been to another country? / Have you never been to another country?	

*1: The contents were determined according to individual biographical background and experimental dates.
